# DPD-YOLO: dense pineapple fruit target detection algorithm in complex environments based on YOLOv8 combined with attention mechanism

**DOI:** 10.3389/fpls.2025.1523552

**Published:** 2025-01-28

**Authors:** Cong Lin, Wencheng Jiang, Weiye Zhao, Lilan Zou, Zhong Xue

**Affiliations:** ^1^ School of Electronics and Information Engineering, Guangdong Ocean University, Zhanjiang, China; ^2^ South Subtropical Crops Research Institute, Chinese Academy of Tropical Agricultural Sciences, Zhanjiang, China

**Keywords:** pineapple detection, UAV, BiFPN, YOLOv8, coordinate attention

## Abstract

With the development of deep learning technology and the widespread application of drones in the agricultural sector, the use of computer vision technology for target detection of pineapples has gradually been recognized as one of the key methods for estimating pineapple yield. When images of pineapple fields are captured by drones, the fruits are often obscured by the pineapple leaf crowns due to their appearance and planting characteristics. Additionally, the background in pineapple fields is relatively complex, and current mainstream target detection algorithms are known to perform poorly in detecting small targets under occlusion conditions in such complex backgrounds. To address these issues, an improved YOLOv8 target detection algorithm, named DPD-YOLO (Dense-Pineapple-Detection YOU Only Look Once), has been proposed for the detection of pineapples in complex environments. The DPD-YOLO model is based on YOLOv8 and introduces the attention mechanism (Coordinate Attention) to enhance the network’s ability to extract features of pineapples in complex backgrounds. Furthermore, the small target detection layer has been fused with BiFPN (Bi-directional Feature Pyramid Network) to strengthen the integration of multi-scale features and enrich the extraction of semantic features. At the same time, the original YOLOv8 detection head has been replaced by the RT-DETR detection head, which incorporates Cross-Attention and Self-Attention mechanisms that improve the model’s detection accuracy. Additionally, Focaler-IoU has been employed to improve CIoU, allowing the network to focus more on small targets. Finally, high-resolution images of the pineapple fields were captured using drones to create a dataset, and extensive experiments were conducted. The results indicate that, compared to existing mainstream target detection models, the proposed DPD-YOLO demonstrated superior detection performance for pineapples in situations where the background is complex and the targets are occluded. The mAP@0.5 reached 62.0%, representing an improvement of 6.6% over the original YOLOv8 algorithm, Precision increased by 2.7%, Recall improved by 13%, and F1-score rose by 10.3%.

## 1 Introduction

China is recognized as one of the leading pineapple producers in the world, with Guangdong Province having the highest output. At present, the harvesting and yield statistics for pineapples are mainly based on manual picking and recording. However, traditional manual labor is not only time-consuming and laborintensive but also prone to errors. In addition, pineapples are densely distributed in the fields, often obscured by leaves, and significant variations in color, size, and shape exist between different varieties, which makes manual detection challenging. With the development of deep learning technologies and the widespread use of drones in agriculture, new methods for crop monitoring have been introduced (Li et al., 2024a; Jumaah et al., 2024). By integrating drones with object detection technology, the automated identification of pineapples and field monitoring has been enabled, which significantly enhances production efficiency, reduces labor costs, and plays a vital role in the mechanization of pineapple cultivation and harvesting.

Traditional methods for crop detection in agricultural fields mainly rely on local features from agricultural images, such as texture, color, and shape, to describe the targets, and classifiers, such as Support Vector Machines (SVM) (Hearst et al., 1998) and K-Nearest Neighbors (KNN) (Taunk et al., 2019), are employed for target classification. For example, Chaivivatrakul et al. (Chaivivatrakul and Dailey, 2014) proposed a texturebased technique for detecting green fruit on plants. This method involves extracting features, calculating descriptors of interest points, classifying interest points using SVM, mapping candidate fruit points, performing morphological closing, and extracting fruit regions. The detection accuracy for pineapples in a single image was achieved at 85%. Christy et al. (Sari et al., 2020) introduced a method for papaya classification based on a Naïve Bayes Classifier and Local Binary Patterns (LBP) features. The input image was first processed by grayscale conversion and image adjustment, then the papaya leaves were divided into nine regions and LBP features were extracted. The Naïve Bayes Classifier was then used for classification, achieving an accuracy of 90% on the test set. However, these traditional methods rely on manually designed features and are tailored to specific working environments, leading to a reduced success rate in more complex natural environments.

Compared to traditional methods, deep learning techniques are widely applied to the target recognition and detection of crop fruits, achieving significant results. Deep learning-based object detection models are generally categorized into two types: one category includes region proposal-based detection algorithms, such as Faster RCNN (Ren et al., 2017) and Mask R-CNN (He et al., 2017), while the other directly transforms the problem of bounding box localization into a regression problem, eliminating the need for candidate boxes, such as SSD (Liu et al., 2016) and YOLO (Redmon et al., 2016). Several studies have been conducted on fruit target recognition in natural environments, with algorithms being developed for the recognition of fruits like apples, corn, and strawberries. For instance, to enable broccoli heads to be detected by a harvesting robot, a Mask R-CNN based on ResNet-50 was used by Blok et al. (Blok et al., 2021) as the instance segmentation network. The network was trained and tested on a broccoli dataset consisting of three varieties, with data augmentation techniques being applied. Similarly, Yue et al. (Yue et al., 2019) proposed an apple detection method in complex environments by adding a boundary-weighted loss function to the original Mask R-CNN network. High-resolution images captured by drones (Lin et al., 2024), along with their ability to perform multiple visits, have enabled large and detailed datasets to be created, attracting the attention of agricultural experts. Gao et al. (Gao et al., 2024) proposed a real-time maize tassel detection method based on Unmanned Aerial Vehicles (UAVs), where maize field images were first collected using drones. The collected data was then combined with an attention mechanism, spatial pyramid pooling, and Atrous convolution to build a UAV remote sensing platform-based YOLOv5-Tassel (YOLOv5-T) model. In addition, the DSE-YOLO network was introduced by Wang et al. (Wang et al., 2022) to enhance the ability to assess the maturity of small strawberries, where pointwise convolution and dilated convolution were utilized to extract various details and semantic features. Lyu et al. (Lyu et al., 2024) proposed an innovative method for the dynamic monitoring and counting of lotus flowers and seed pods using an improved version of YOLOv7-tiny for unmanned aerial vehicles (UAVs). In their work, they enhanced YOLOv7-tiny by investigating the fusion mechanism between SPD layers and convolutions, leading to the development of a novel SPD-Conv block that surpasses the traditional SPDConv. Additionally, a set of convolutional attention modules was embedded at the neck region to optimize the utilization of channel and spatial information. The modified YOLOv7-tiny model demonstrated a notable improvement, with increases of 3.5%, 0.65%, and 4.05% in map@50, precision (P), and recall (R), respectively, thereby enabling the efficient measurement of lotus flowers and seed pods in super-resolution aerial images.

In recent years, object detection models have also been applied by researchers to the detection and recognition of pineapples in agricultural fields (Lai et al., 2023; Yu et al., 2023; Cuong et al., 2022). For example, an improved YOLOv3 model has been proposed by Liu et al. (Liu et al., 2023) to locate pineapples by combining stereo vision with the enhanced YOLOv3 model. Challenges such as insufficient lighting, strong exposure, and severe occlusion encountered in field based pineapple detection have been addressed by Meng et al. (Meng et al., 2023) through the use of a shift window transformer, which integrates a region based convolutional neural network. This approach captured long distance pineapple images to increase the number of instances, achieving an accuracy of up to 92.54%. A lightweight pineapple detection model based on YOLOv7 was introduced by Zhang et al (Zhang et al., 2023), which accelerated the detection speed. The model parameters were significantly reduced by Li et al. (Li et al., 2024b) through pruning and lightweighting of the backbone, enabling the deployment of the pineapple detection algorithm on agricultural robots with limited computational power. However, existing model designs do not adequately focus on small targets. In high altitude overhead scenes, pineapples are often obstructed by branches or heavily occluded by one another, making the task of detecting densely packed small targets challenging. The detection performance of these models often fails to meet the requirements, and real time detection is prevented due to slow detection speed.

To address the aforementioned issues, a high-resolution pineapple field image dataset has been collected and created using drones in this paper. A novel object detection algorithm, named DPD-YOLO (DensePineapple-Detection You Only Look Once), has been proposed specifically for pineapple detection tasks in complex environments. Extensive experiments conducted on the pineapple dataset have demonstrated that, compared to existing mainstream object detection models, DPD-YOLO exhibits highly competitive detection performance, particularly in complex backgrounds and when the targets are occluded.

Overall, the contributions of this paper are summarized as follows:

1. To address the challenges of dense targets and difficult feature extraction, a small target P2 detection layer has been introduced into the network to capture target information at different scales, thereby improving the accuracy of small target detection. To mitigate feature information loss caused by consecutive convolutions, a Weighted Bi-directional Feature Pyramid Network (BiFPN) has been employed to fuse deep semantic information with shallow spatial information, enhancing the model’s generalization capability and enabling the network to effectively capture multi-scale features, thus improving its ability to perceive high-density targets.
2. In response to the challenges posed by the close color similarity between pineapple leaves and crowns, as well as the obstruction of fruits by leaves in the background, an attention mechanism known as Coordinate Attention has been introduced into the YOLOv8 network. This enhancement increases the network’s focus on the critical feature information of small pineapple targets, suppresses interference from irrelevant background information, and improves the feature extraction capability for small targets within the detection network.
3. Small targets in complex backgrounds are considered difficult samples, posing significant challenges to model detection. To enhance detection accuracy, the original YOLOv8 network integrates the Decoder from RT-DETR as its detection head. The Decoder processes the output features from the encoder using Self-Attention and Cross-Attention mechanisms, allowing more spatial location information of targets to be obtained and improving the model’s ability to handle overlapping or interrelated targets.
4. Currently, mainstream loss functions pay limited attention to difficult samples. In the context of UAVcaptured pineapple fields, where the size of pineapples is generally small, the CIoU loss function has been modified by introducing Focaler-IoU to enhance the network’s focus on difficult samples in the image.

The remainder of the paper is organized as follows. In Section 2, the image acquisition process and the creation of the dataset are introduced, and a detailed description of the methodological contributions is provided. In Section 3, the experimental results are presented, along with a comprehensive performance analysis and comparisons with mainstream methods. Section 4 concludes the paper.

## 2 Materials and methods

### 2.1 Dataset

**Figure 1:**
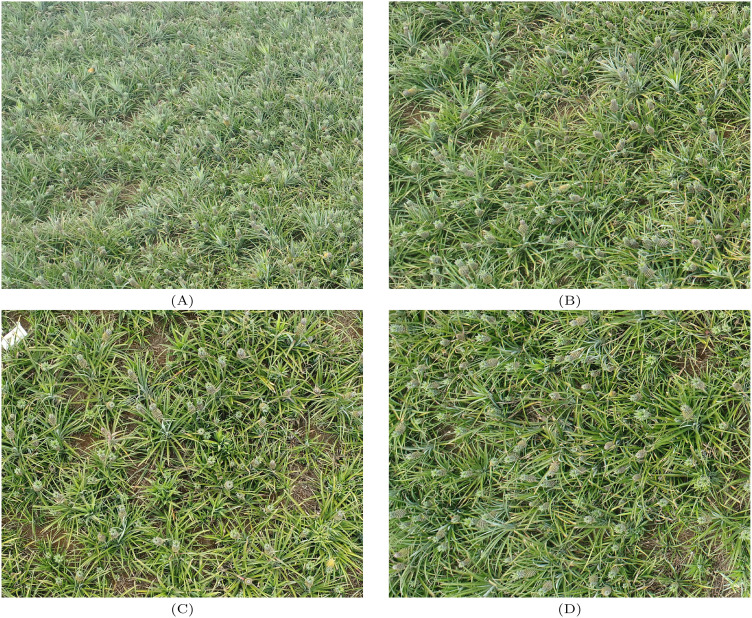
**(A–D)** represent some of the images in this article’s data set.

The images in this dataset were collected on September 25, 2022, in Xuwen County, Zhanjiang City, Guangdong Province, China. This county is primarily dedicated to pineapple cultivation and benefits from a tropical monsoon humid climate, characterized by relatively high temperatures and abundant rainfall throughout the year. The dry season is marked by ample sunshine, which contributes to the high yield and quality of pineapples in the region. The images were captured using a DJI Mavic 3 Pro drone equipped with a 4/3 CMOS sensor, resulting in each image having a resolution of 5472×3078. The drone operated at an altitude ranging from 125 to 135 meters, and the dataset contains a total of 153 images with 32,371 labeled targets. The dataset was randomly divided into training, validation, and test sets in an 8:1:1 ratio. Some of the images in the dataset are shown in **Figure 1**. The annotation software used for this dataset is LabelImg, and the annotation work was guided by experts from both the agricultural field and the computer vision field. The dataset was manually annotated, with the pineapples in the images labeled to ensure that the bounding boxes completely enclosed the pineapples and distinguished them from the background. After each image was annotated, an XML file was generated, storing the coordinates of the rectangular bounding boxes for each labeled pineapple in the image. However, the XML files could not be directly used for YOLOv8 training, so the data had to be converted to YOLO format. For each image, the category and bounding box coordinates for each pineapple were stored in a separate text file. Each record in the file corresponded to one bounding box in the image, including the center pixel coordinates of the annotated object, and the size of the bounding box was represented by the coordinates of the top-left and bottom-right corners.

### 2.2 Methods

#### 2.2.1 YOLOv8

The YOLOv8 object detection algorithm is one of the YOLO series algorithms developed by the Ultralytics team. Compared with previous YOLO algorithms, the innovations presented in this paper were primarily reflected in the following aspects.

**Figure 2:**
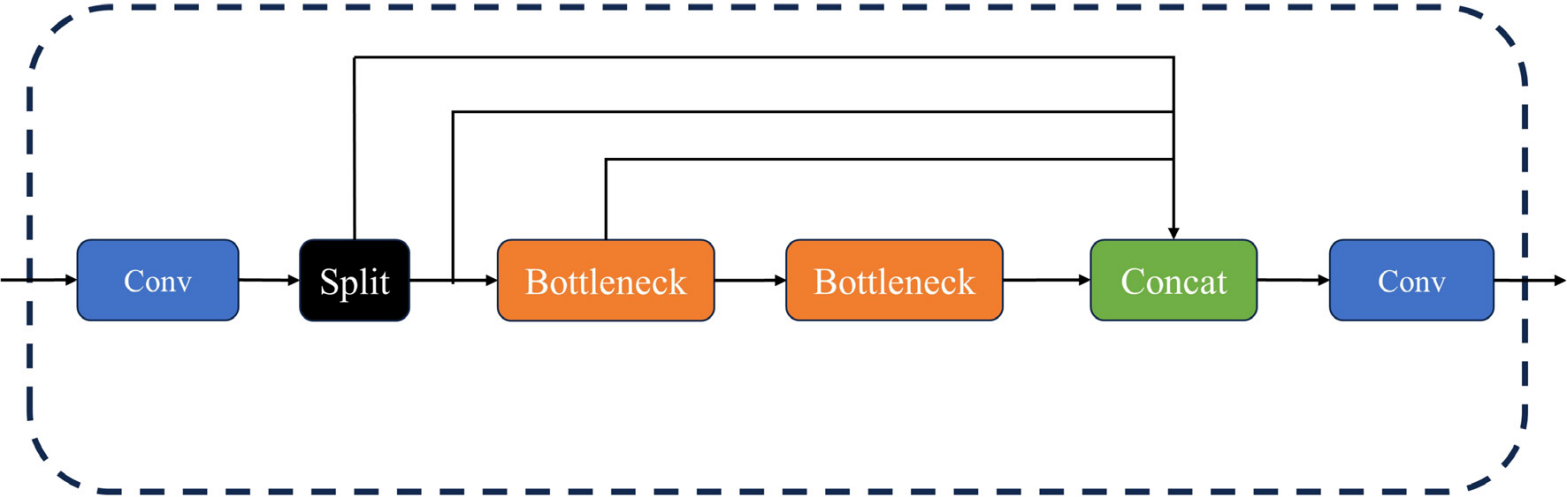
The C2f structure diagram.

- A new network module, named C2f, was proposed for use in the backbone network of YOLOv8. It consists of both Conv and C2f modules. Within the C2f module, additional skip layer connections were added, convolution operations in the branches were eliminated, and an additional split operation was introduced. The above design not only retains rich feature information but also effectively reduces the computational load. A schematic diagram of the C2f structure is shown in **Figure 2**.
- In this paper, the Anchor-Based method was changed to an Anchor-free method. The main difference between the two lies in whether predefined anchor boxes are used to match the ground truth boxes. An anchor box is a set of preset bounding boxes designed to assist in training the predicted boxes (Prediction box) to offset their positions relative to the ground truth boxes (Ground truth box). The essence of this mechanism is to address the issue of label assignment. Anchor boxes are used not only to locate the positions of the bounding boxes but also to define their sizes and shapes, in conjunction with the key points extracted from the feature map by the network.

The loss used during training is the Binary Cross-Entropy Loss (BCE Loss), and its calculation formula is provided as follows:


(1) $$\begin{array}{c} Loss_{BCE}=-\frac{1}{N}\sum_{i=1}^{N}[y_{i}\text{ log}(p_{i})+(1-y_{i})\text{ log}(1-p_{i})] \end{array}$$

where *N* is denoted as the total number of samples. *yi* is the true label for the *i*-th sample, and *pi* is the predicted probability of the *i*-th sample belonging to the positive class.

DFL (Distribution Focal Loss) is designed to allow the network to quickly focus on values near the labels, thereby maximizing the probability density at the label locations. The cross-entropy function is employed in DFL to optimize the probabilities at positions adjacent to the labels, ensuring that the network’s predictions are concentrated around the label values. The formula is provided as follows:


(2) $$\text{DFL }(S_{i},S_{i+1})=-((y_{i+1}-y)\text{ log}(S_{i})+(y-y_{i})\text{ log }(S_{i+1})).$$

where *S_i_* and *S_i_*_+1_ are denoted as the model-predicted probabilities for the *i*-th and (*i* + 1)-th discrete intervals, respectively. Here, *y* is taken as the true continuous-valued target, while *y_i_* and *y_i_*_+1_ are defined as the boundary values that define the *i*-th and (*i* + 1)-th intervals. The terms $\left(y_{i+1}-y\right)$ and $\left(y-y_{i}\right)$ represent the relative proximity of *y* to the upper boundary *y_i_*_+1_ and the lower boundary *yi*, respectively. As *y* approwaches *y_i_*, the value of $\left(y_{i+1}-y\right)$ increases, and the influence of the *i*-th interval is emphasized. Conversely, as *y* approaches $y_{i+1}$ , $\left(y-y_{i}\right)$ becomes larger, and the emphasis shifts toward the (*i* + 1)-th interval.

The regression loss function used in YOLOv8 is CIoU, and the calculation formula is given as follows:


(3) $${\text{Loss}}_{CIoU}=1-CIoU,$$

(4) $$\text{CIoU }=IoU-\frac{\rho^{2}(B,B^{gt})}{c^{2}}-\alpha v,$$

(5) $$\alpha =\frac{v}{1-IoU+v},$$

(6) $$v=\frac{4}{\pi^{2}}{\left({\text{tan}}^{-1}\frac{w^{gt}}{h^{gt}}-r{\text{tan}}^{-1}\frac{w}{h}\right)}^{2},$$

where *IoU* is defined as the ratio of the intersection area to the union area between the predicted box and the ground truth box. It is used to measure the degree of overlap between the predicted and ground truth boxes. *B* is denoted as the center point of the predicted box, and *B^gt^* is denoted as the center point of the ground truth box. *ρ*(*B, B^gt^*) is defined as the distance between the center points of the predicted and ground truth boxes. *c* represents the diagonal length of the smallest enclosing region that can contain both the predicted and ground truth boxes. *α* is a balancing parameter that does not participate in the gradient calculation. *v* is used to represent the aspect ratio between the predicted and ground truth boxes, and *w^gt^* and *h^gt^* are defined as the width and height of the ground truth box, respectively. *w* and *h* are defined as the width and height of the predicted box, respectively. When the predicted and ground truth boxes have equal aspect ratios, the value of *v* is 0.

**Figure 3:**
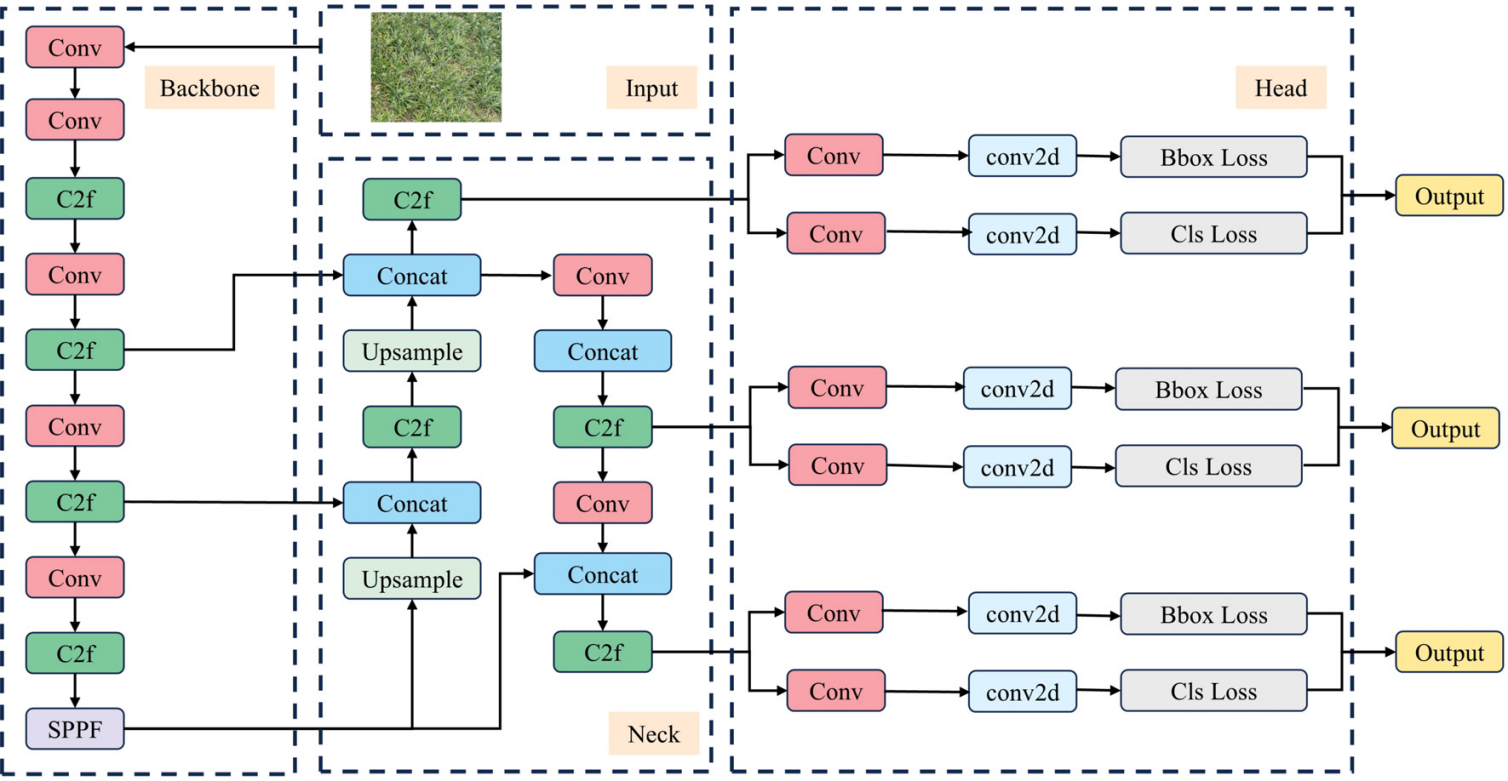
YOLOv8 structure diagram.

- The positive sample matching strategy is based on the TaskAlignedAssigner assignment strategy, which uses the weighted scores from both classification and regression to select positive samples. The formula is given as follows:

(7) $$t=s^{\alpha }*u^{\beta },$$

where *α* and *β* are weight hyper-parameters, *s* represents the score of the predicted category, and *u* is the Intersection over Union (IoU) between the predicted box and the ground truth box. The product of these two values is used to effectively measure the matching degree. When both the classification score and the IoU are high, the value of *t* approaches 1, indicating a stronger match between the predicted box and the ground truth box, thereby aligning more closely with the positive sample criteria. For each ground truth box, the matching degrees are sorted, and the top *K* predicted boxes are selected as positive samples. When a single predicted box is matched with multiple ground truth boxes, the ground truth box with the highest IoU is retained. A schematic diagram of the YOLOv8 network structure is shown in **Figure 3**.

#### 2.2.2 P2+BiFPN

The original YOLOv8 network is composed of three detection heads, designed for small, medium, and large-scale object detection. However, for small object detection, these targets are often characterized by limited features. As the convolutional neural network deepens, important information tends to be lost in the feature maps after convolution operations. In the dataset used in this paper, the pineapples captured by drones are considered relatively small, which leads to suboptimal training results when the original YOLOv8 is used. Therefore, a small object detection layer, P2, is proposed to be added to the YOLOv8 network to enhance its ability to detect pineapples.

YOLOv8 is employed with a Path Aggregation Network-Feature Pyramid Network (PAN-FPN). The design of PAN is aimed at enhancing feature representation by integrating feature information from different receptive fields. However, its effectiveness in handling multi-scale features is limited. The Feature Pyramid Network (FPN) is specifically designed to improve object detection tasks by performing feature fusion across multiple resolution levels, thereby enhancing the ability to detect objects of various scales. However, traditional FPNs use a top-down feature fusion method, which may result in the loss of object location information. To address these two issues, PAN-FPN combines PAN and FPN, constructing a network structure that flows both from the bottom up and from the top down. This structure achieves feature fusion of shallow location information and deep semantic information, ensuring feature diversity and completeness.

**Figure 4:**
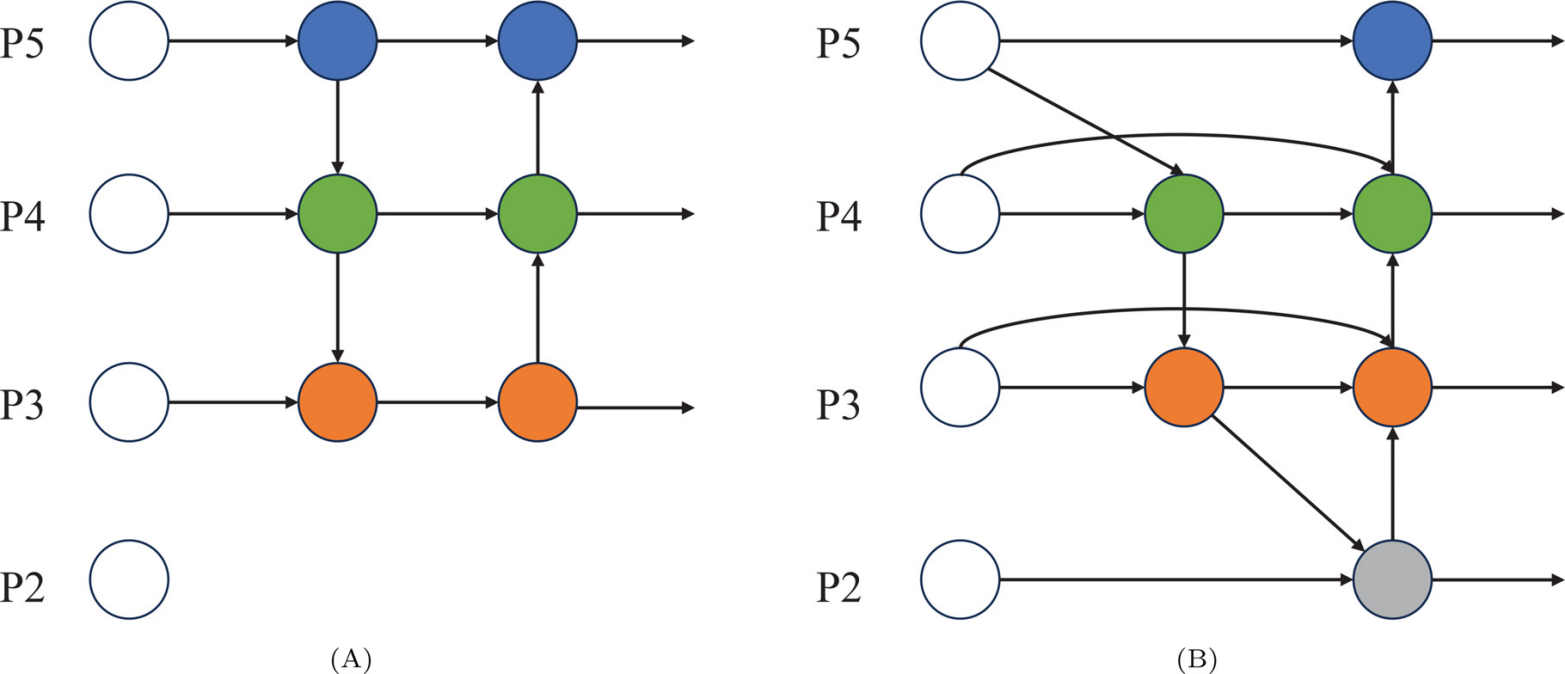
**(A)** represents the network structure of PAN-FPN and **(B)** represents the network structure of BiFPN.

The BiFPN (Bi-directional Feature Pyramid Network) (Tan et al., 2020), proposed by Tan et al., is widely employed in most object detection models to perform multi-level feature extraction from input images using convolutional neural networks. Deep networks are characterized by large receptive fields and strong semantic representation capabilities, but their feature maps tend to have lower resolutions and lack spatial details. In contrast, shallow networks are known for having higher feature map resolutions and rich details, but they are characterized by weaker semantic expression capabilities. Therefore, the detection capability of the network is improved by fusing features across different levels. To address the issue of inadequate feature fusion in PANet, cross-level multi-scale fusion is introduced by BiFPN, with the calculation formulas for layers P3 and P4 provided as follows:


(8) $$P_{3}^{td}=\frac{Conv((\omega_{1}\cdot P_{3}^{in}+\omega_{2}\cdot \text{Resize}(P_{4}^{in})))}{\left(\omega_{1}+\omega_{2}+\epsilon \right)},$$

(9) $$P_{3}^{out}=\frac{Conv((\omega_{1}^{'}\cdot P_{3}^{in}+\omega_{2}^{'}\cdot P_{3}^{td}+\omega_{3}^{'}\cdot \text{Resize}(P_{4}^{out})))}{\left(\omega_{1}^{'}+\omega_{2}^{'}+\omega_{3}^{'}+\epsilon \right)},$$

where $P_{3}^{td}$ is the intermediate feature at the third level of the top-down path, $P_{3}^{in}$ represents the feature extracted from the third layer of the backbone network, and $P_{3}^{out}$ denotes the output feature at the fourth level of the bottom-up path. The weights ω and $\omega_{0}$ are assigned to each feature map, while *ϵ* is treated as a constant. All other features are constructed in a similar manner. The BiFPN structure is illustrated in **Figure 4**.

#### 2.2.3 Coordinate attention

In computer vision, the attention mechanism is designed to mimic the ability of the human visual system to focus on salient parts of complex scenes. When an image is input to the model, the attention mechanism dynamically allocates weights based on the importance of different regions in the image (Guo et al., 2022). CA (Coordinate Attention) (Hou et al., 2021), proposed by Hou et al., is a novel mobile network attention mechanism that integrates positional information into channel attention. Two one-dimensional global pooling operations are employed in Coordinate Attention to merge input features into two independent directional feature maps—one for the vertical direction and the other for the horizontal direction. These feature maps, which contain direction-specific information, are encoded into two mappings, each of which captures the longrange dependencies of the input feature map in a specific spatial direction. Positional information is preserved in the generated mappings, and the two attention maps are multiplied and applied to the input feature map.

**Figure 5:**
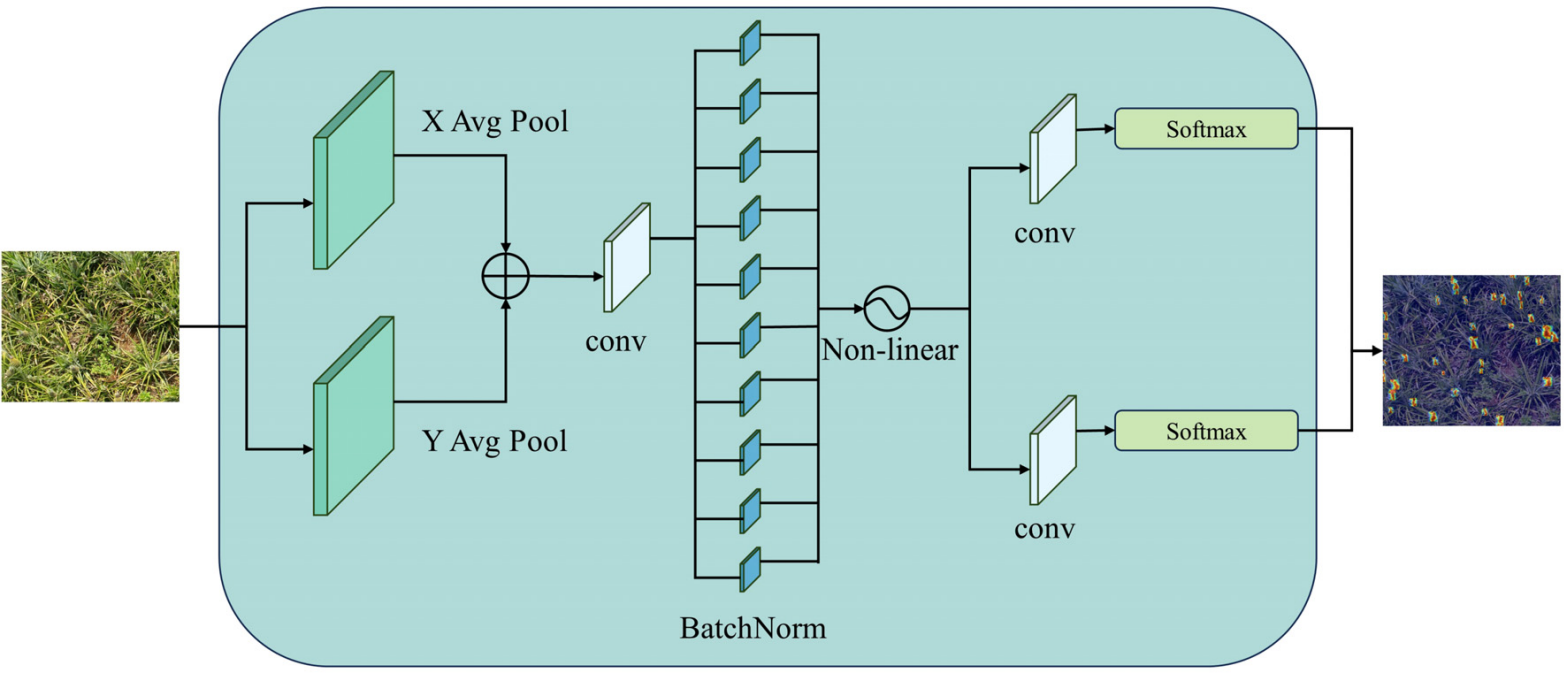
Coordinate attention structure diagram.

The structure diagram of Coordinate Attention is shown in **Figure 5**.

#### 2.2.4 RT-DETR head

RT-DETR (Real-Time Detection Transformer) (Zhao et al., 2023b) combines the advantages of the Transformer architecture, providing an efficient end-to-end solution for object detection. The head structure of RT-DETR is primarily composed of an encoder and a decoder. In the encoder, the Efficient Hybrid Encoder is used to transform multi-scale features into a sequence of image features through intra-level feature interaction (AIFI) and cross-scale feature fusion modules (CCFM). Additionally, IoU-aware query selection is employed, where a fixed number of image features are selected as the initial object queries for the decoder, allowing the model to focus on the most relevant targets in the scene, thereby enhancing detection accuracy.

The decoder is considered one of the cores of RT-DETR, with the output features from the encoder being processed using self-attention and cross-attention mechanisms. Within the decoder, the self-attention mechanism allows the information from other queries to be considered by each query, aiding in the handling of overlapping or interrelated targets. The cross-attention mechanism enables interaction between object queries and the feature maps from the encoder, helping the spatial location information of the targets to be learned.

**Figure 6:**
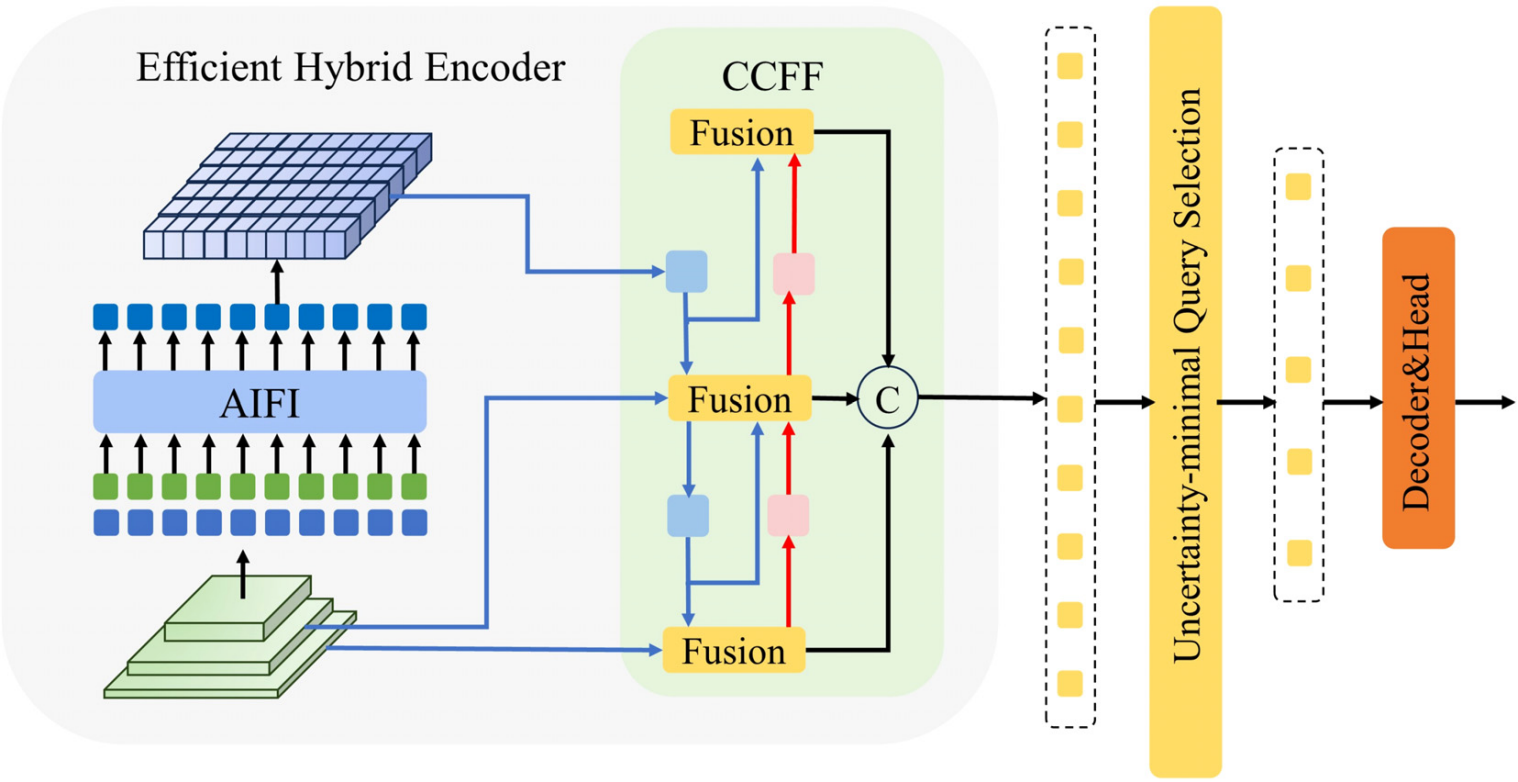
The RT-DETR head structure diagram.

A schematic diagram of the RT-DETR head structure is shown in **Figure 6**.

#### 2.2.5 Focaler-CIoU

In object detection tasks, the issue of sample imbalance is commonly encountered. Based on the size of the detected objects, samples are generally categorized into easy samples and hard samples. Easy samples typically possess more common sizes, while smaller targets are usually classified as hard samples due to challenges in feature extraction and localization. It has been argued by Zhang et al. (Zhang and Zhang, 2024) that focusing on the bounding box regression process of easy samples is beneficial for improving detection performance in tasks dominated by easy samples. In contrast, special attention must be given to the bounding box regression hard samples in tasks dominated by them. Therefore, Focaler-IoU has been proposed, which enhances the performance of detectors in various detection tasks by focusing on different types of regression samples. To effectively address targets of different sizes, a linear interval mapping method has been employed by Zhang et al. to reconstruct the IoU loss, thereby enhancing bounding box regression capabilities. The formula is as follows:


(10) $$IoI_{focaler}=\{\begin{array}{c} 0,IoU<d\\ \frac{IoU-d}{u-d},d\ll IoU\ll u\\ 1,IoU>u, \end{array}$$

where $IoU_{focaler}$ is defined as the reconstructed IoU, while *IoU* represents the normally calculated IoU value, with [*d,u*] ∈ [0,1]. By adjusting the values of *d* and *u*, different types of regression samples can be focused on by the Focaler-IoU model. The calculation formula for Focaler-IoU is shown as follows:


(11) $$Loss_{Focaler-IoU}=1-IoU_{focaler}.$$

Focaler-IoU can be applied to various types of IoU. In this paper, CIoU is improved using Focaler-IoU, and the calculation formula for the enhanced Focaler-CIoU is provided as follows:


(12) $$Loss_{Focaler-CIoU}=Loss_{CIoU}+IoU-IoU_{Focaler}.$$

#### 2.2.6 DPD-YOLO

**Figure 7:**
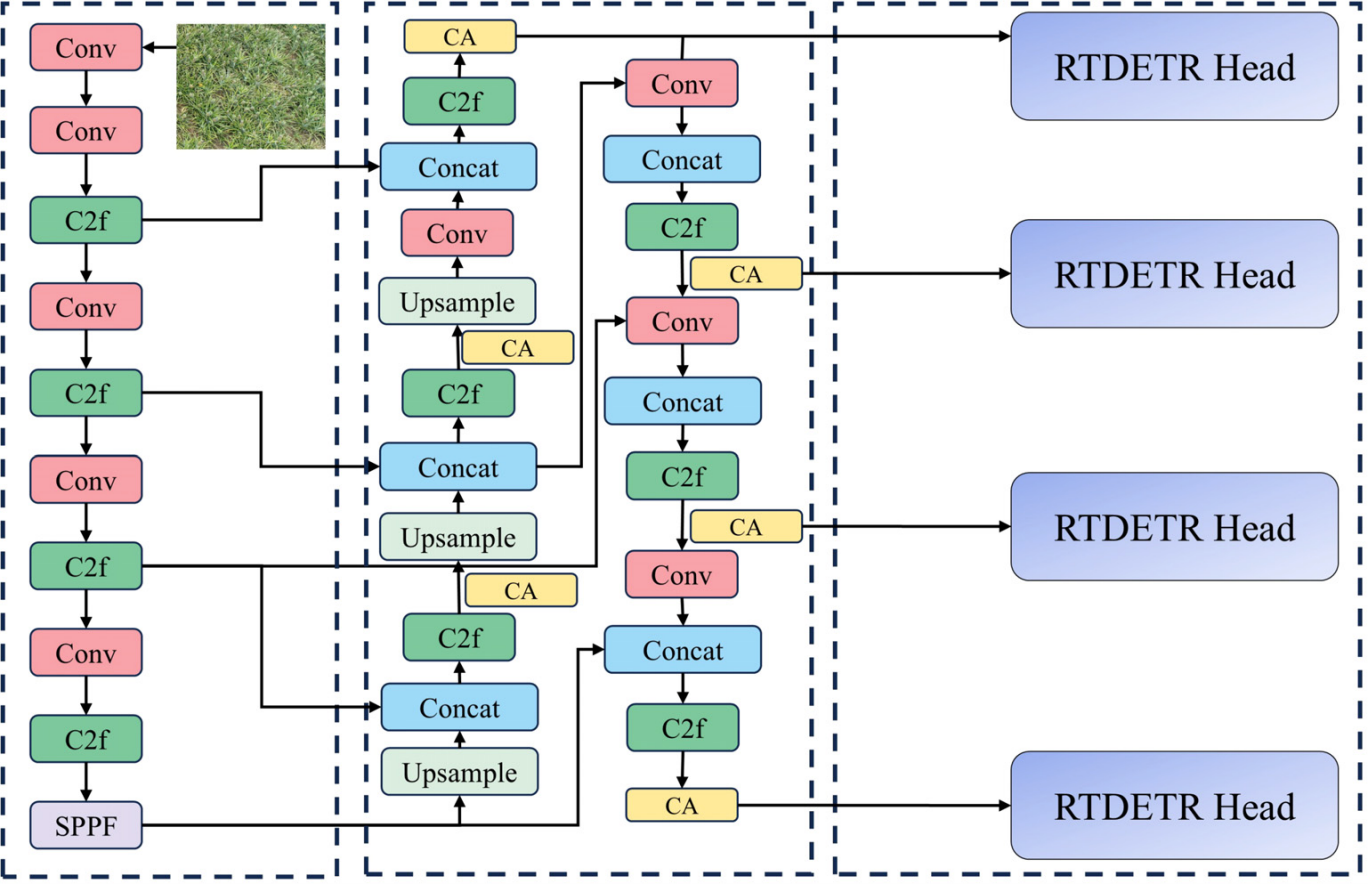
DPD-YOLO structure diagram.

The network structure of DPD-YOLO is shown in **Figure 7**. Structural improvements have been introduced in DPD-YOLO while maintaining the overall architecture of YOLOv8. Key innovations in DPD-YOLO include the addition of a small target detection layer (P2), which increases the number of detection scales from three to four, allowing smaller targets to be focused on and improving detection performance for small objects.

Additionally, the PAN-FPN (Path Aggregation Network with Feature Pyramid Network) used in YOLOv8 has been replaced with BiFPN, which integrates the large receptive field and strong semantic information representation of deeper layers with the high-resolution, feature-rich characteristics of shallow layers. This fusion of features across different layers has been shown to enhance detection capabilities. Furthermore, a Coordinate Attention mechanism has been incorporated into DPD-YOLO, allowing the model to focus more on targets and reducing the influence of complex backgrounds on detection performance. Moreover, a regression function based on an improved CIoU version of Focaler-CIoU has been employed, enhancing detection performance by focusing on different regression samples and improving the ability to detect densely packed pineapple targets. Finally, the detection head in DPD-YOLO has been replaced with the RT-DETR Head. This module uses an encoder to transform multi-scale features, and a decoder with self-attention mechanisms is utilized to handle overlapping or related targets. The cross-attention mechanism in the decoder interacts with the feature map to extract spatial location information of the targets. In summary, a series of improvements have been proposed by DPD-YOLO to enhance the detection of dense pineapple fruits in complex backgrounds, addressing the challenges encountered by YOLOv8 in detecting small and densely packed targets.

### 2.3 Model training and testing

#### 2.3.1 Training processing platform

During training, the learning rate and other hyperparameters are set based on a comprehensive evaluation of model performance and experimental experience. To ensure that sufficient time is allowed for the model to learn and optimize, the training epochs are set to 300. The initial learning rate is set to 0.01 to provide a moderate step size for parameter updates, enabling the model to learn features quickly during the initial stages training while avoiding oscillations or convergence failure that may be caused by excessively large step sizes (Sirisha et al., 2023). To enhance the generalization ability and stability of the model, a weight decay term with a value of 0.0005 has been added. This helps to reduce the risk of overfitting by limiting the growth of model parameters. Furthermore, Stochastic Gradient Descent (SGD) is employed for model optimization, which is efficient for handling large-scale datasets. A momentum term of 0.937 is introduced to retain information about the previous gradient direction during parameter updates, thus accelerating convergence and reducing oscillations in the updating process. The design of these hyperparameters fully considers the efficiency and stability of model training, ensuring that the model can effectively address target detection tasks in complex backgrounds. In this study, the input image size for the YOLOv8 network is set to 640∗640. The effect of different input image sizes on model accuracy is demonstrated in the official code repository. When the input image size is 1280∗1280, the model accuracy is higher compared to when the input size is 640∗640. However, as the input image size increases, the computational resources required for model training and inference also increase, leading to longer processing times. The images in the dataset used in this study are of high resolution, with a size of 5472∗3078, and the pineapples are densely packed in the images. Given these factors, the input image size is set to 640∗640. Regarding kernel size, different convolutional kernel sizes correspond to different receptive field sizes on the input feature maps. By stacking multiple small kernels, a receptive field equivalent to that of large kernels can be achieved, while using smaller kernels reduces the number of parameters and computational load. In YOLOv8, the kernel size is set to 3∗3. A comparison experiment with different kernel sizes was conducted, and the results are presented in **Table 1**. From **Table 1**, it can be observed that a kernel size of 3∗3 produces the best performance.

**Table 1:** Impact of different kernel sizes on model performance.

Method	Kernels	P	R	F1	mAP@0.5	Size (MB)
YOLOv8	1∗1	72.3%	28.0%	40.4%	25.1%	17.5
YOLOv8	3∗3	83.5%	49.0%	61.8%	55.4%	21.4
YOLOv8	5∗5	82.4%	51.0%	63.0%	54.5%	29.3
DPD-YOLO	1∗1	83.2%	33.0%	47.3%	32.8%	16.7
DPD-YOLO	3∗3	86.2%	62.0%	72.1%	62.0%	20.1
DPD-YOLO	5∗5	84.8%	61.0%	71.0%	62.0%	29.3

#### 2.3.2 Evaluation index

The performance of the deep learning model is evaluated after training using specific metrics to determine whether improvements are needed for optimal performance (Ye et al., 2024). Common evaluation metrics in object detection tasks, such as Precision, Recall (Everingham et al., 2010), mean Average Precision (mAP@0.5), and F1-score (Lin et al., 2014), are primarily selected for model evaluation in this paper. Precision is defined as the proportion of true positives among detected targets, reflecting the accuracy of detection. Recall is the number of correctly detected targets among all image samples, indicating the completeness of the model in target detection. F1-score, a metric used in binary classification models, combines both Precision and Recall. A score closer to 1 is considered to indicate better performance. Average Precision (AP) (Chowdhury, 2010) is the area under the Precision-Recall curve, reflecting the effectiveness of the model in recognizing a specific category. Mean Average Precision (mAP@0.5) is the average of AP values across all categories and is used as an indicator of the overall performance of the model. True Positive (TP) refers to instances where the model predicts a positive sample that matches the actual positive sample. False Positive (FP) refers to instances where the model predicts a positive sample, but the actual sample is negative. False Negative (FN) refers to instances where the model predicts a negative sample, while the actual sample is positive. True Negative (TN) refers to instances where the model predicts a negative sample that corresponds to the actual negative sample. The calculation of *Precision* is presented in the following formula:


(13) $$Precision=\frac{TP}{TP+FP}\times 100\%.$$

The calculation of Recall is presented in the following formula:


(14) $$Recall=\frac{TP}{TP+FN}\times 100\%.$$

The calculation of F1 -score is presented in the following formula:


(15) $$F1=\frac{2\times Precision\times Recall}{Precision+Recall}\times 100\%.$$

The calculation of AP is presented in the following formula:


(16) $$AP=\int_{0}^{1}P(R)dR\times 100\%.$$

The calculation of mAP is presented in the following formula:


(17) $$mAP=\frac{\sum_{i=1}^{N}AP_{i}}{N}.$$

## 3 Experimental results and analysis

### 3.1 Ablation experiment

Under the same experimental platform and test set conditions, it was found that the baseline model, without improvement strategies, achieved a Precision of 83.5%, a Recall of 49.0%, and a mean Average Precision (mAP@0.5) of 55.4%. After the RT-DETR Head was switched in, the Precision of the model was increased to 84.0%, the Recall was raised to 58.0%, and the mAP@0.5 was achieved at 56.1% indicating that the RTDETR Head had a significant effect on enhancing detection capability. After the addition of BiFPN and the integration of small target detection layers, the Precision of the model was further improved to 85.6%, while the Recall remained at 61.0%, and the mAP@0.5 was elevated to 58.2%. This indicates that the integration of small target detection layers within BiFPN significantly enhanced the ability of the network to extract target features. Finally, after the Coordinate Attention mechanism was incorporated, it was found that a Precision 85.6%, a Recall of 62.0%, and a mAP@0.5 of 61.9% were achieved by the model. This demonstrates that the YOLOv8 model, enhanced by the RT-DETR Head, the integrated small target detection layers in BiFPN, and the addition of the Coordinate Attention mechanism, exhibited significant improvements in target feature extraction and detection capabilities, with a Precision increase of 2.1%, a Recall increase of 13.0% and a mAP@0.5 increase of 6.5% compared to the baseline model. The size of the baseline model was recorded as 21.4 MB. Although an increase in model size was observed following the substitution of detection heads, a notable reduction of 19.7% in model size was achieved after the BiFPN and small target detection layers were incorporated. The model size, after the addition of the Coordinate Attention mechanism, remained 1.3 MB lower than that of the baseline model, indicating that the integration of small target detection layers within BiFPN effectively optimized model size. Additionally, after CIoU was improved to Focaler-CIoU, enhancements in Precision, F1-score, and mean Average Precision (mAP@0.5) were observed without negatively impacting model size. The experimental results are presented in **Table 2**.

**Table 2:** Ablation experiments.

DETR-Head	P2+BiFPN	CA	Focaler-CIoU	P	R	F1	mAP@0.5	Size (MB)
×	×	×	×	83.5%	49.0%	61.8%	55.4%	21.4
✓	×	×	×	84.0%	58.0%	68.6%	56.1%	24.9
✓	✓	×	×	85.6%	61.0%	71.2%	58.2%	20.0
✓	✓	✓	×	85.6%	62.0%	72.0%	61.9%	20.1
✓	✓	✓	✓	86.2%	62.0%	72.1%	62.0%	20.1

✓ indicates that this improvement is used; × indicates that this improvement is not used.

Currently, various types of attention mechanisms are present in the field of computer vision, but not every mechanism is capable of positively impacting model performance. Therefore, multiple mechanisms were compared during the selection process, and EMA (Efficient Multi-Scale Attention), CPCA (Channel Prior Convolutional Attention), SimAM (Simple Attention Module), MLCA (Mixed Local Channel Attention), and Coordinate Attention were chosen for experimentation. EMA is an efficient multi-scale attention mechanism that converts some channels into batch dimensions to preserve the information of each channel while reducing the consumption of computational resources. The channel weights of each parallel branch are calibrated by encoding global information, and cross-dimensional aggregation of the output features from the two parallel branches is performed. CPCA combines channel attention and spatial attention, aiming to enhance feature representation and dynamically allocate attention weights. Spatial information is effectively extracted through multi-scale depthwise separable convolutions, while channel priors are retained. SimAM, based on neuroscientific theories, proposes a method to optimize the energy function to evaluate the importance of each neuron, thereby inferring three-dimensional attention weights for feature maps without increasing the original network parameters. MLCA is a lightweight mixed local channel attention mechanism that improves detection accuracy by combining channel information and spatial information simultaneously. The experimental results are presented in **Table 3**.

**Table 3:** Comparison of the effects of different attention mechanisms.

Attention	P	R	F1	mAP@0.5	Size(MB)
EMA	85.2%	59.0%	69.8%	57.2%	20.1
CPCA	85.9%	55.0%	67.0%	54.5%	20.5
SimAM	86.5%	58.0%	69.4%	55.8%	20.0
MLCA	87.8%	60.0%	71.2%	57.0%	20.1
Coordinate Attention	86.2%	62.0%	72.1%	62.0%	20.1

**Figure 8:**
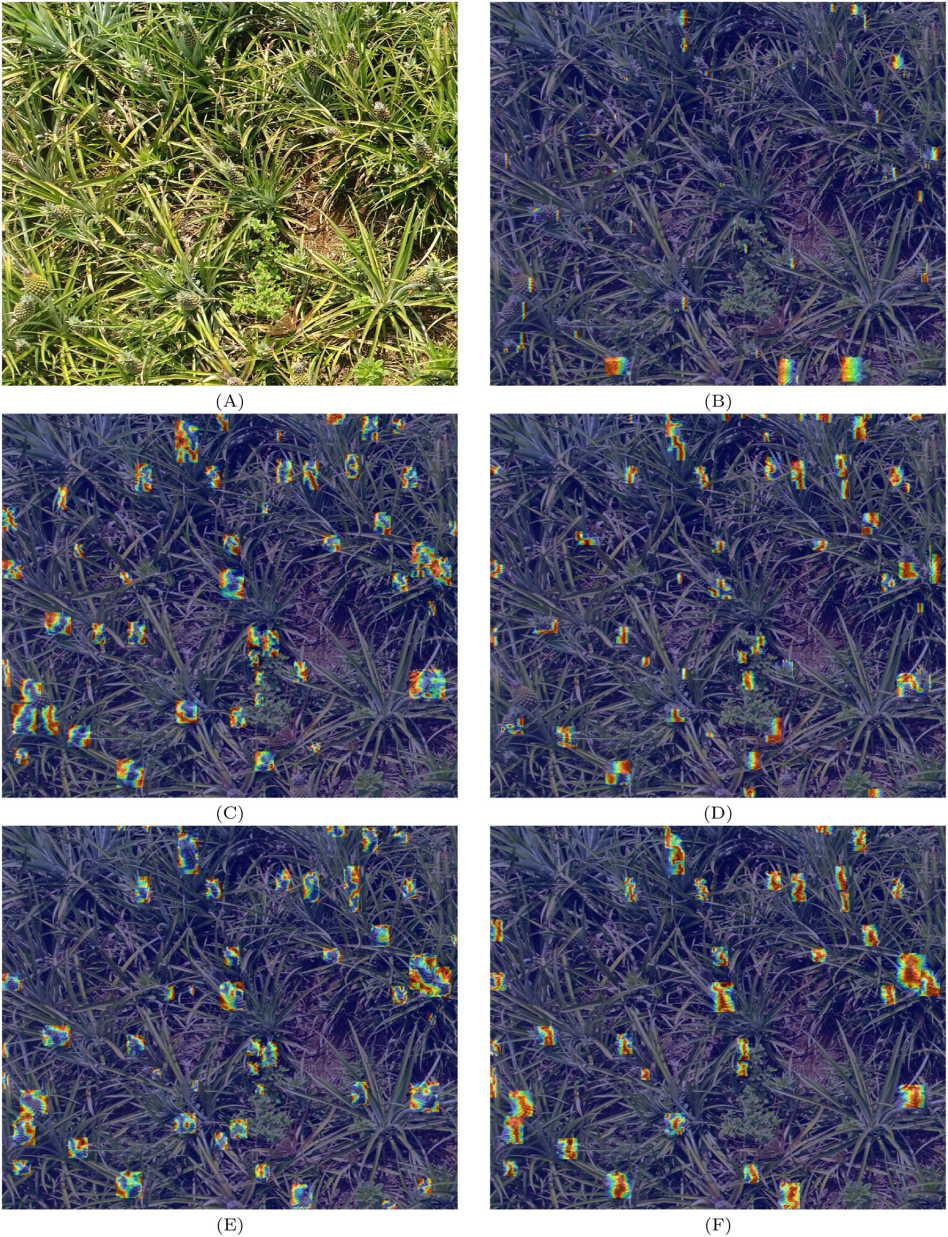
**(A)** represents the original image, **(B–F)**, in turn, represents the effect of EMA, CPCA, SimAM, MLCA, and Coordinate Attention.

To more intuitively demonstrate the effects of attention mechanisms, Grad-CAM (Selvaraju et al., 2017) was adopted to output feature maps for visualizing the heat maps. The heat maps generated by different attention mechanisms at the output layer of the YOLOv8 network are shown in **Figure 8**. **Figure 8A** represents the original image, while **Figures 8B–F** represent the heat maps after the application of EMA, CPCA, SimAM, MLCA, and Coordinate Attention, respectively. In the heat maps, areas with redder colors indicate higher attention from the model toward those areas. From **Figure 8**, it can be observed that the YOLOv8 model, with the introduction of EMA and SimAM in **Figures 8B, D**, shows lower attention toward the pineapple in the image, with fewer markings on the heat map. Although **Figures 8C, E** have more marked regions, their effectiveness in focusing on the pineapple is not ideal. In contrast, **Figure 8F**, while having fewer marked areas, demonstrates significantly higher attention toward the pineapple, with most marked regions effectively focusing on it.

### 3.2 Comparison of loss values

**Figure 9:**
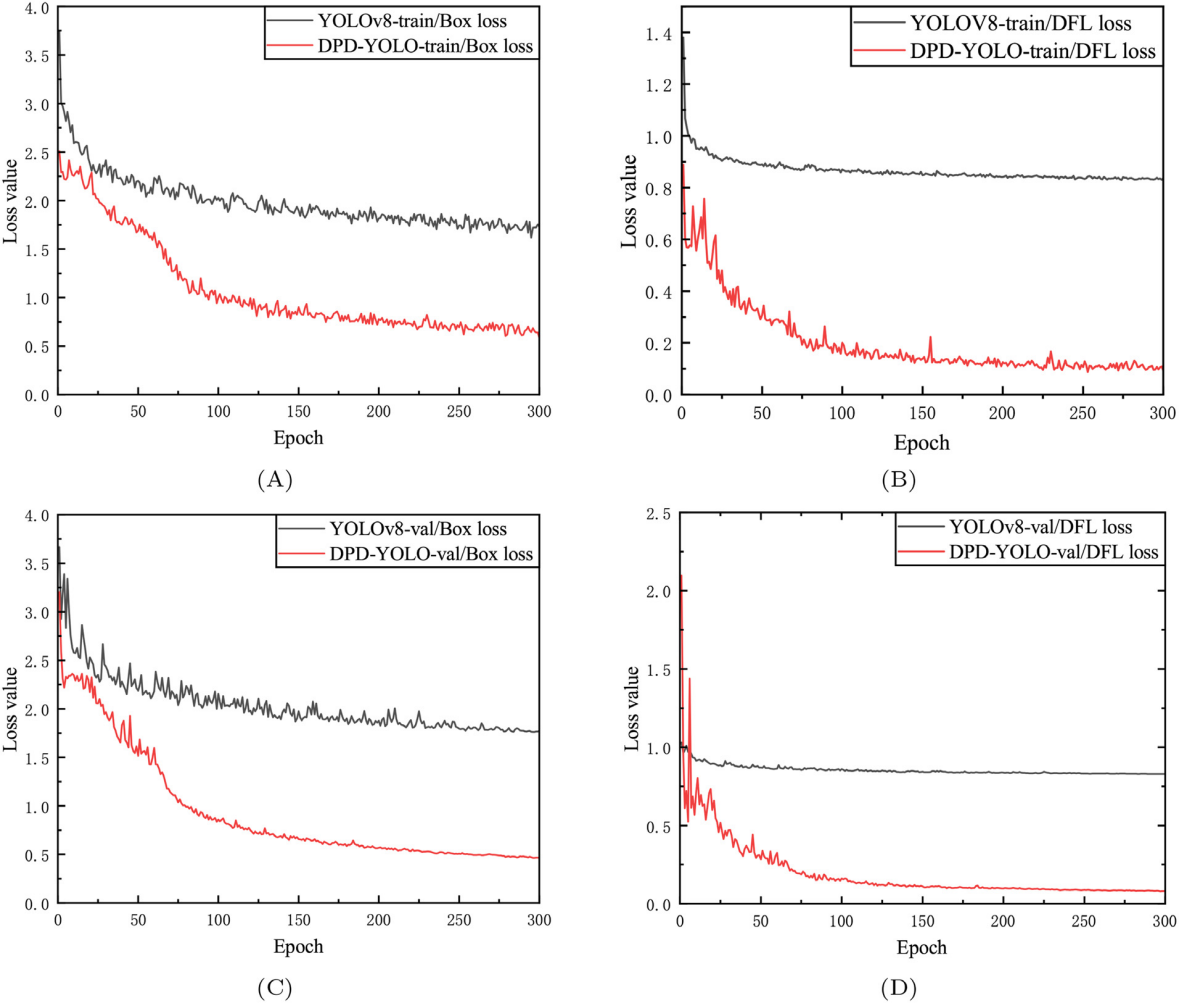
Training validation results. **(A)** represents the Box Loss curve of YOLOv8 and DPD-YOLO training, **(B)** represents the DFL Loss curve of YOLOv8 and DPD-YOLO training, **(C)** represents the Box Loss curve verified by YOLOv8 and DPD-YOLO, **(D)** represents the DFL Loss curve verified by YOLOv8 and DPD-YOLO.

**Figure 9** shows the DFL loss and box loss generated by DPD-YOLO and YOLOv8 during training and validation.

The x-axis represents the epoch, and the y-axis represents the loss value. During training, both the training and validation loss values of the model are observed to decrease as the number of epochs increases, eventually stabilizing (Zhao et al., 2023a). From the figure, it can be seen that the DFL loss and Box loss of DPD-YOLO on the validation set are both found to be lower than those of YOLOv8 on the same set, which indirectly indicates that a stronger learning ability is exhibited by DPD-YOLO in scenarios with dense pineapples compared to YOLOv8.

### 3.3 Comparison of different detection algorithm

To validate the superiority of the proposed DPD-YOLO in detecting pineapples from a drone perspective, DPD-YOLO was compared with other classic object detection algorithms, including Centernet, Faster R-CNN, SSD, RetinaNet, and the YOLO series versions v3, v5, X, v7, v8, v9, and v10. The comparison results are presented in **Table 4**.

**Table 4:** Performance comparison of different algorithms.

Method	P	R	F1	mAP@0.5	Size (MB)
Centernet	85.5%	8.9%	16.1%	27.3%	124
Faster R-CNN	19.7%	4.75%	7.7%	2.0%	521
SSD	6.9%	0.2%	0.4%	0.7%	90.6
Retainnet	86.8%	0.7%	1.4%	1.56%	138
YOLOv3	86.9%	51.0%	64.3%	51.7%	198
YOLOv5s	83.3%	56.0%	67.0%	57.2%	13.7
YOLO-X	89.9%	41.8%	57.1%	57.6%	34.3
YOLOv7	64.8%	2.3%	4.5%	17.1%	142
YOLOv8	83.5%	49.0%	61.8%	55.4%	21.4
YOLOv9	84.2%	54.0%	65.8%	57.0%	19.3
YOLOv10	80.8%	43.0%	56.1%	42.0%	15.7
DPD-YOLO	86.2%	62%	72.1%	62.0%	20.1

The red font in the table represents the DPD-YOLO model proposed in this paper and its related experimental results.

From **Table 4**, it can be seen that the highest Recall rate, mAP@0.5 value, and F1-score are achieved by the proposed DPD-YOLO, with a model size of 20.1MB, compared to other classic object detection algorithms. Centernet is based on an anchor-free strategy, where each target is represented by the center point of the object; however, relatively low Recall rate and mAP@0.5 values are observed in the pineapple detection task. Feature extraction, candidate box generation, localization, and classification are integrated in Faster R-CNN, but lower detection accuracy is demonstrated on the dataset used in this study. As one-stage algorithms, both SSD and RetinaNet are found to show suboptimal detection accuracy in the complex pineapple field environment. Among the algorithms in the YOLO series, YOLOv3, YOLOv5, YOLO-X, YOLOv7, YOLOv9, and YOLOv10 are found to exceed 50% detection accuracy on the dataset used in this study, with the exceptions of YOLOv7 and YOLOv10. However, the larger model size of YOLOv3 is found to affect its feasibility for transfer and deployment. Although YOLOv5 has the smallest model size, lower F1-score and mAP@0.5 values are observed compared to the proposed DPD-YOLO. Overall, the experimental results indicate that the proposed DPDYOLO outperforms other classic object detection algorithms in detection performance, and good practicality is demonstrated.

**Figure 10:**
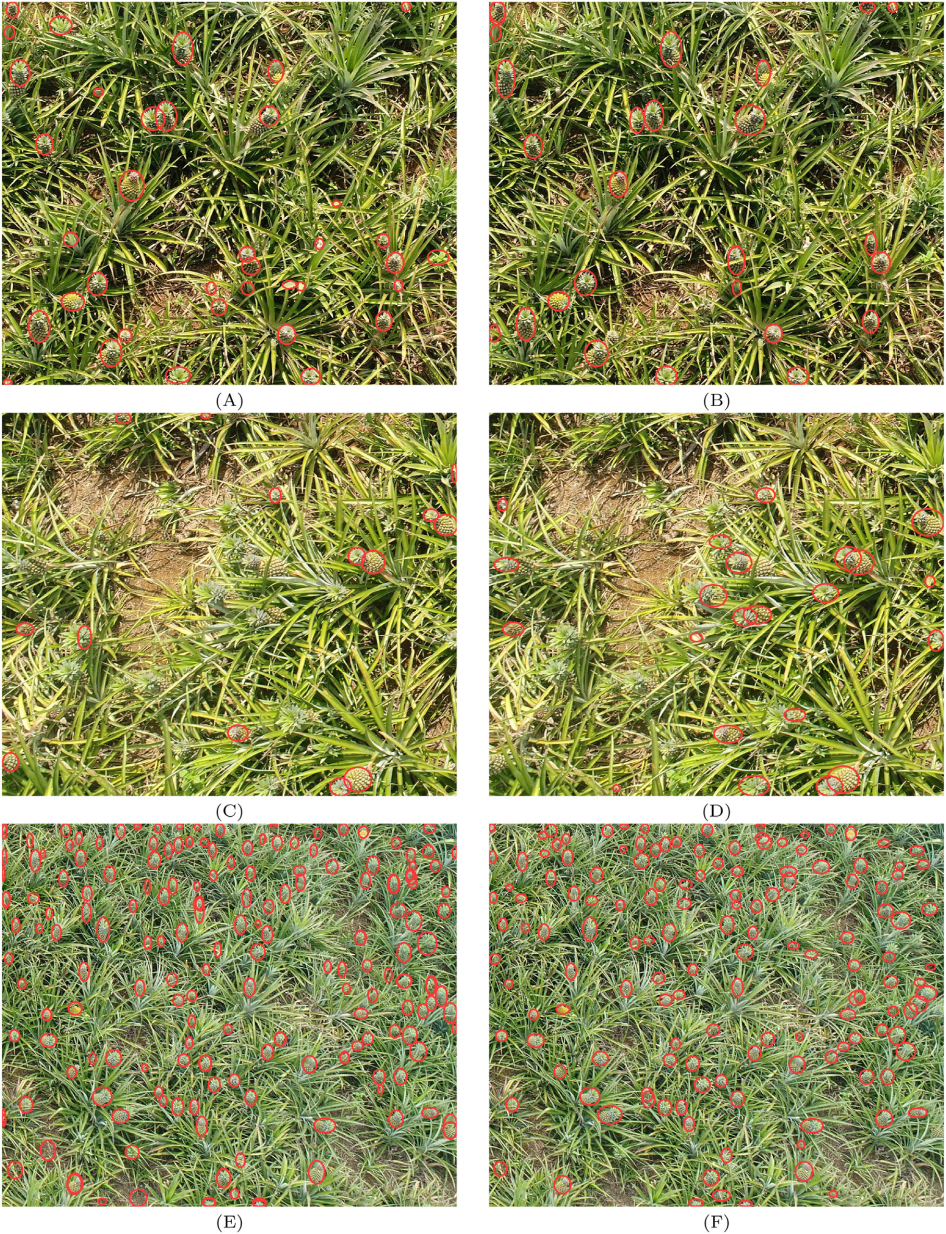
**(A, C, E)** represent the detection results of YOLOv8 before improvements, **(B, D, F)** show the results of DPD-YOLO.

In **Figures 10A, C, E** represent the detection results of YOLOv8 before improvements, while **Figures 10B, D, F** show the results after improvements. A comparison reveals that a considerable number of false positives and missed detections are exhibited by the original YOLOv8 when identifying pineapples. In contrast, significant improvements in detection performance are shown by the improved YOLOv8 in the same areas, with both false positives and missed detections being effectively reduced. The detection results of YOLOv8 on drone images of pineapples before and after the improvements are illustrated in **Figure 10**. Before the enhancements, numerous false positives and missed detections were encountered by YOLOv8 when faced with complex backgrounds and occlusions, particularly when pineapples were partially obscured by other plants, which significantly impacted detection accuracy. After the improvements, a marked enhancement in YOLOv8’s detection performance was observed, especially in handling occlusion scenarios. More effective recognition of pineapples, even when partially covered, was achieved by the improved model. Greater robustness was exhibited by the improved model, with high detection accuracy being maintained in complex environments, thereby enhancing target recognition capabilities. It is indicated that the DPD-YOLO algorithm has a clear advantage in handling dense small targets and complex backgrounds.

## 4 Conclusions

A novel algorithm named DPD-YOLO is proposed in this study, which has been designed to address the challenges of detecting pineapples in aerial images of pineapple fields captured by drones, where targets are small, densely distributed, and heavily occluded. Based on YOLOv8, the BiFPN module with smallobject detection layers has been integrated to enhance the model’s ability to fuse features of small targets. Additionally, the Coordinate Attention mechanism has been introduced, and Focaler IoU has been employed to improve the traditional CIoU, enabling a more accurate focus on pineapple targets in complex backgrounds. Furthermore, the original detection head has been replaced with RT-DETR, which utilizes self-attention and cross-attention mechanisms to capture spatial positional information of the targets more effectively. Experimental results have been presented, demonstrating that DPD-YOLO has outperformed existing stateof-the-art object detection methods across multiple evaluation metrics such as Recall, F1, and mAP@0.5, showcasing exceptional performance. Notably, the method has been shown to possess remarkable capability in accurately identifying pineapples, even in complex and occluded backgrounds, thereby confirming the robustness and efficacy of the model.

However, the current dataset is relatively limited, particularly in terms of coverage under different environmental conditions. Therefore, future research should focus on the expansion of the dataset to include data from various lighting conditions and weather scenarios, ensuring that pineapple targets can be effectively detected by DPD-YOLO in diverse and complex environments, thus further enhancing the robustness and practicality of the model. Additionally, further optimization of the network architecture is needed to reduce the model size and the number of parameters, which will result in improved performance for practical applications, while meeting the requirements for real-time efficiency and high performance.

## Data Availability

The raw data supporting the conclusions of this article will be made available by the authors, without undue reservation.
